# X-ray histology on rapidly fixed fresh tumor tissue samples for fast resection margin assessment

**DOI:** 10.1038/s41598-026-61069-6

**Published:** 2026-07-11

**Authors:** Jenny Romell, Carlos Fernandez Moro, Bertha Brodin, Laszlo Szekely, Edvin Porovic, Panagiotis Tsagkozis, Ernesto Sparrelid, Mikael Björnstedt, Hans M. Hertz

**Affiliations:** 1 Dept. of Applied Physics, KTH Royal Inst of Technology/Albanova, 10691 Stockholm, Sweden; 2 Excillum AB, Torsnästorget 17, 164 40 Kista, Sweden; 3 Department of Clinical Pathology and Cancer Diagnostics, F46 Karolinska University Hospital, 141 86 Stockholm, Sweden; 4 Department of Laboratory Medicine, Division of Pathology, Karolinska Institute, Stockholm, Sweden; 5 Department of Acute and Reparative Medicine, Karolinska University Hospital, Stockholm, Sweden; 6 Division of Surgery and Oncology, Department of Clinical Science, Intervention and Technology, Karolinska Institute, Karolinska University Hospital, Stockholm, Sweden

**Keywords:** X-ray histology, Phase contrast, Resection margin assessment, Cancer, Engineering, Medical research, Oncology

## Abstract

Accurate assessment of the resection margin is of key importance for patient outcome after tumor surgery. Intraoperative assessment is preferable but present methods like fresh-frozen sectioning risk sampling errors and provide only 2D information. Classical 2D histology has better quality but is too slow for intraoperative feedback. In the present proof-of-concept study we show that laboratory x-ray histology based on propagation-based phase-contrast microtomography has potential for 3D intraoperative resection margin assessment. Our system includes a liquid–metal-jet high-brightness microfocus source, precision stages and a high-resolution detector, providing fast, high-contrast, near-cellular-resolution 3D imaging. The tissue is prepared by rapid acetone fixation before the 3D x-ray imaging. We demonstrate the method on several tumor types in liver and pancreas, as well as on different sarcomas. After the x-ray histology, classical histology was performed on the same samples for comparison. The two methods compare excellently on the liver and pancreas samples and reasonably on the morphologically more difficult sarcomas. The imaging currently takes 4–5 h with our prototype instrument, and we outline how to go below 1 h for the full procedure. We conclude that 3D x-ray histology can be developed to provide intraoperative resection margin assessment.

**Supplementary Information:**

The online version contains supplementary material available at 10.1038/s41598-026-61069-6.

## Introduction

Surgery is an essential part of the curative plan for most cancer patients with solid tumors. The outcome of such surgery (i.e., recurrence rates and ultimately patient survival) depends on several factors where the resection margin is a key indicator. The margin assessment is either performed post-operatively with classical histology, which provides great detail but takes several days, and/or intraoperatively, which often has lower quality but allows for immediate corrective surgery. Both methods are typically 2D. Here we propose a method for 3D intraoperative resection margin assessment based on rapid fixation/dehydration and fast cellular-resolution phase-contrast x-ray microtomography.

Numerous studies have consistently shown that margin status is a key indicator for patient outcome for many common cancers^1–3^. Therefore, each surgery is followed by routine histopathology assessing the closest distance (in mm) between the tumor cells and the cut edge. This work-intensive process includes fixation, slicing, selection, multi-step dehydration, infiltration, embedding, sectioning, rehydration, and staining. Such classical histology provides good accuracy and pathological insight via elaborate staining methods in combination with high-resolution 2D microscopy. However, it does not provide 3D data and is slow (typically several days), making the decision to perform corrective surgery non-trivial since it entails an additional procedure usually after resubmission of the patient. Furthermore, the orientation of the areas with questionable radicality is often difficult.

For the most common solid cancers, classical histopathology finds tumor-positive cells in the resection margin in 10–35% of the cases^4^. For less common cancers the number increases to 40–60%^5^. Furthermore, due to the present process of slicing, the pathologist risks missing small groups of tumor cells hidden within the part of the paraffin block that is not sectioned. Inadequate margins can lead to re-excision surgery, but such operations need to be weighed against patient risk and discomfort. As an alternative, the post-surgery treatment plan is often adjusted, including chemotherapy and/or radiation therapy. The high rates of positive margins clearly motivate better methods for intraoperative feedback enabling immediate corrective surgery, both from a patient outcome perspective and from a health-economy perspective.

Presently the only methods for intraoperative guidance in standard use are fresh-frozen sectioning and 2D radiography^6^. Fresh-frozen sectioning typically requires 0.5–1 h and is only feasible on a limited amount of tissue. Its reliability varies due to the risk of sampling errors and the quality of the histological section is generally lower than in classical histopathology. Different implementations of 2D radiography are used during breast-conserving surgery (BCS) to allow for removal of cancer from the breast while leaving as much as the normal tissue as possible. A wealth of other methods have been evaluated with the aim to provide intraoperative assessment^5,6^. These include imaging techniques like spectroscopy (hyperspectral, Raman, mid-IR etc.), fluorescence (confocal, two-photon, light sheet etc.), structured illumination (OCT, SHG), and opto-acoustics, as well as classical pre-operative radiology (x-ray, micro-CT, MRI, ultrasound). None has to date been successful, with the exception of intraoperative micro-calcification assessment in breast surgery evaluated by classical x-rays.

Phase-contrast x-ray microtomography allows 3D x-ray imaging of low-atomic-number materials like soft tissue, where classical absorption contrast does not suffice^7,8^. For intraoperative resection margin assessment, high spatial resolution and short exposure times are essential. This requires a high-brightness source and consequently early x-ray histology was performed at synchrotron sources. The liquid–metal-jet electron-impact microfocus source^9^ operates at an order of magnitude higher brightness than competing laboratory sources and has potential for another order of magnitude (http://www.excillum.com). This small-spot and high-power x-ray tube enables phase-contrast x-ray imaging with high spatial resolution^10^ and adequate exposure times in the clinic.

Phase-contrast x-ray tomography on excised samples was first suggested as alternative to conventional histology by Zanette et al.^11^. The advantages include high speed, 3D imaging with thinner effective slicing, and less destructive and simplified sample preparation. Such x-ray histology has been demonstrated at synchrotron radiation facilities^11–14^, and with laboratory sources^15,16^. It has since been extended to tumor imaging and resection margin assessment, for breast tumors at synchrotrons with high resolution^17^ and in the laboratory with lower resolution^18^, as well as for upper abdominal cancers with high resolution^19^. For our goal of intraoperative feedback, high speed and high resolution are necessary in addition to local accessibility, ruling out the synchrotron-source-based methods.

For completeness, we note that the use of staining agents for improved absorption tomography has been producing high-resolution high-quality images in x-ray histology and pathology^20–22^. However, the introduction of these stains into tissue is typically too slow for our end goal, intraoperative assessment.

Here we present a proof-of-concept study describing the essential steps towards intraoperative resection margin assessment. The method is based on fast cellular-resolution propagation-based phase-contrast x-ray microtomography on rapidly acetone-fixed tissue samples^23^. We demonstrate our method on samples from common cancers in upper abdomen (liver and pancreas) as well as different sarcomas. The total time is presently a few (3–6) hours of fixation followed by 4–5 h imaging and image processing. Finally, we outline how to go below one hour for the full procedure.

## Results

### Experimental design

**Fig. 1:**
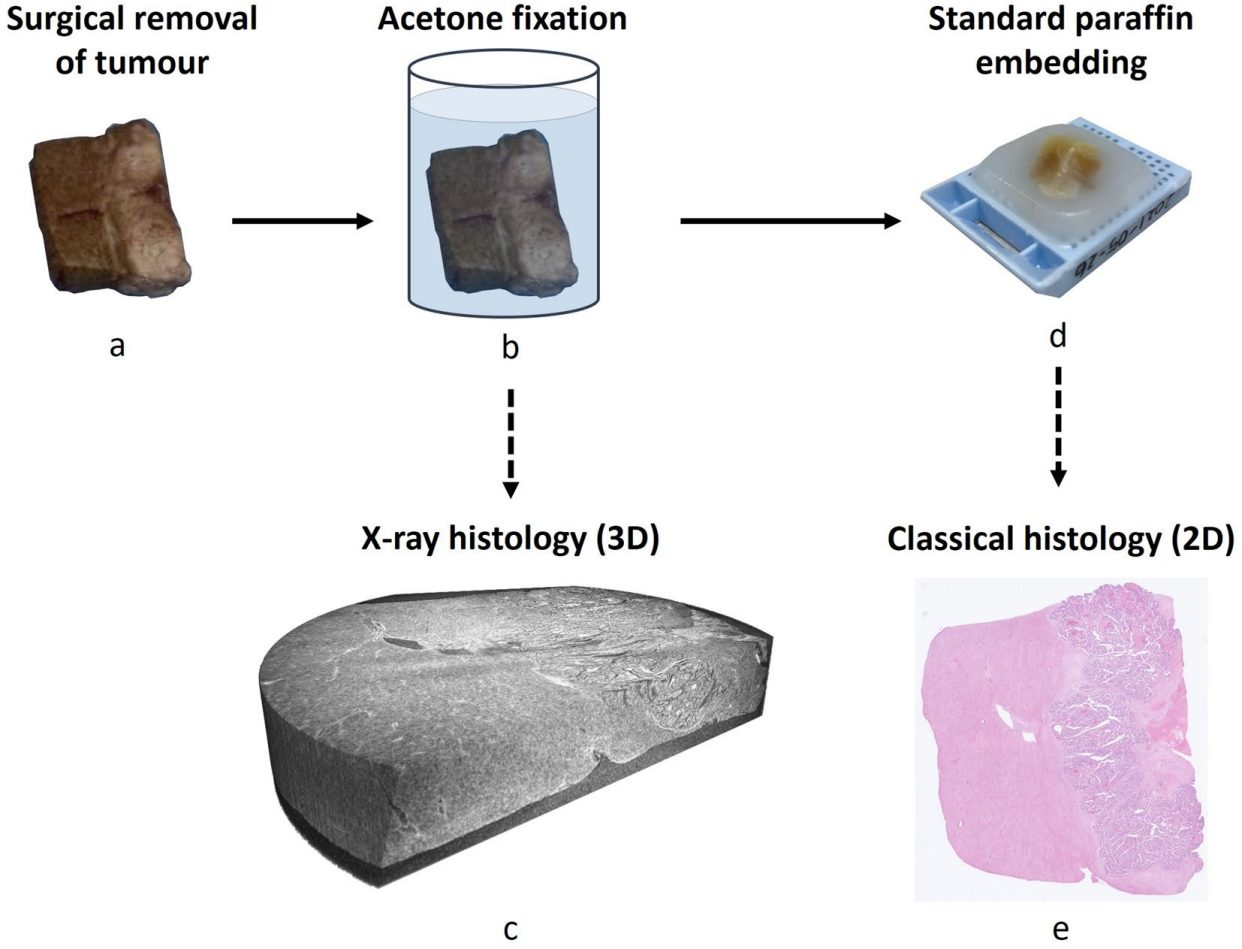
Workflow for x-ray and classical histology of solid tumor. (**a**) The tumor, including a margin of healthy tissue, is surgically removed from the patient and sliced in ~ 5 mm slabs. (**b**) Fixation in 100% acetone. (**c**) 3D x-ray histology by phase-contrast microtomography. (**d**) Standard histological sample preparation, including formalin fixation, dehydration, paraffin embedding, sectioning and staining. (**e**) Light microscopy of H&E-stained slice.

Figure 1 shows the imaging workflow. The full analysis is done on a sample of tumor and adjacent non-tumorous tissue obtained from the fresh resection specimen, ex vivo, shortly after surgery. The key steps are rapid fixation/dehydration in acetone, fast high-resolution phase-contrast microtomography, and image processing and visualization. Finally, we compare the x-ray histology with classical histology on the same samples. Below we outline the steps. More details can be found in “Materials and methods”.

#### Rapid acetone fixation/dehydration

A sample of the fresh tumor from the surgery is placed in 100% acetone. A typical sample size is 5 × 25 × 25 mm (liver, pancreas) as well as smaller diagnostic biopsies (sarcomas). Acetone is known for its faster penetration of tissue compared to ethanol and methanol. By exchanging the classical formaldehyde fixation step followed by a series of dehydration steps in increasing concentration of alcohol for a single-step acetone fixation/dehydration step we can reduce the preparation time from a few days to a few hours before the sample is ready for imaging. Depending on tissue type, the morphology may be slightly more affected compared to when using the gentler method of gradually increasing alcohol concentrations. However, for the purpose here (i.e., to determine if the resection margin is free of tumor cells) this limitation is not a major concern: the morphological changes are relatively small, allowing reliable assessment of the tumor border. Tissue shrinkage is discussed in more detail in the Supplementary Material.

#### Phase-contrast x-ray microtomography

The fixed and dehydrated sample is placed in the laboratory propagation-based phase-contrast imaging arrangement (cf. Fig. S1)^15,16,19^. It consists of an x-ray source (125W, 70 kV microfocus liquid–metal-jet source), a precision rotation stage and a 4096 × 4096 pixel CMOS detector with a 10 µm Gadox scintillator. Imaging is performed with 2 × geometric magnification, resulting in a pixel resolution of 4.5 µm and a FWHM blur of 11 µm. The effective distance z_eff_ was adjusted to obtain maximum contrast for cellular-sized structures^15^, while still keeping the exposure times reasonable (2–3 h) with the present low-power source.

#### Image processing and visualization

This includes initial image corrections, phase-retrieval, tomographic reconstruction, different artifacts and noise reduction routines, and finally displaying the full 3D volume. From this 3D volume virtual histology slices are extracted. Typical processing time is 1–2 h.

#### Classical histology

After the x-ray imaging, the acetone-fixed samples were fixed in formaldehyde 4% buffered solution, embedded in paraffin, sectioned and stained with hematoxylin and eosin (H&E) according to standard procedure, after which a classical histopathology assessment was performed by a specialized pathologist.

#### Comparison

Finally, the x-ray histology and the classical histology of the same sample were compared. Due to the different states of the sample in the two imaging modalities (fixed tissue slab versus micrometer-thin slice), the classical histology slice does not exactly overlap with the virtual x-ray tomography slice, but it is close enough for a relevant comparison.

The method has been investigated for several types of tumors. Below we present results for increasingly difficult tumor morphologies, from liver over pancreas to sarcomas.

### Case series

A total of 12 tumors from 12 individual patients were analyzed, comprising surgical resection specimens of colorectal liver metastases (n = 3), hepatocellular carcinoma (n = 3), intrahepatic cholangiocarcinoma (n = 1), pancreatic neuroendocrine tumor (n = 1), pancreatic ductal adenocarcinoma (n = 1), and diagnostic biopsy probes of chondrosarcoma (n = 1), soft-tissue sarcoma (n = 1), and osteosarcoma (n = 1). Below we present results from 2 types of liver cancer, 2 types of pancreatic cancer and 2 types of sarcomas. Data from all remaining samples is available in Supplementary Material (Figs. S2–S7). Full 3D stacks are available on request.

### Liver tumors

Here we show the results for two common types of liver tumors: Colorectal liver metastasis (n = 3) and hepatocellular carcinoma (n = 3). Intrahepatic cholangiocarcinoma (n = 1) is presented in the Supplementary Material (Fig. S4).

**Fig. 2:**
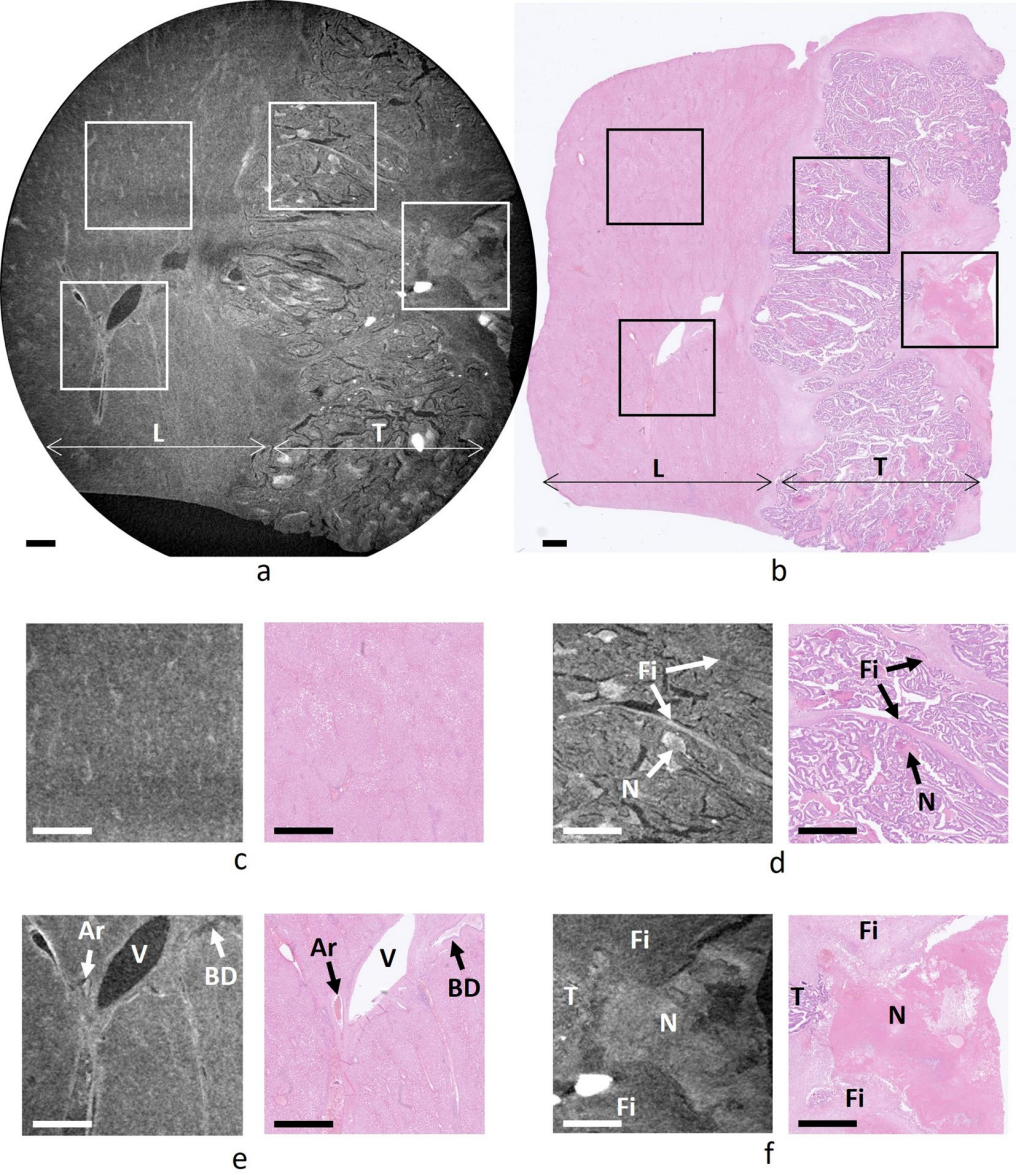
Colorectal liver metastasis. (**a**) Maximum-intensity projection (MIP) virtual slice through the microtomography volume, matching (**b**) the classical histology slice (H&E staining). The sample contains normal liver (L) and tumor (T). (**c**) Liver tissue, as seen with x-ray histology (left) and classical histology (right). (**d**) Tumor periphery, with fibrous septa (Fi) and necrotic foci (N). (**e**) Portal zone, with vein (V), artery (Ar) and bile duct (BD). (**f**) Tumor (T), necrosis (N) and fibrosis (Fi) in the central region of the metastasis. Scale bars: 1 mm.

Figure 2 compares the x-ray histology and the classical histology for the colorectal liver metastasis. Colorectal cancers are the second most deadly cancer type^24^. Approximately 30% of the patients affected develop the liver metastasis investigated here.

Figure 2a,b show a virtual slice of the phase-contrast x-ray microtomography (a) that matches the classical histology slice (b). The tumor tissue (right) is easily distinguishable from the normal liver tissue (left). In Fig. 2c the normal liver tissue is shown for the two methods. The appearance is similar but with somewhat larger graininess in the x-ray histology. Figure 2d shows the tumor periphery for the two methods. In addition to its characteristic large glandular pattern, fibrotic septa and foci of necrosis are observable with both methods. Figure 2e shows a medium-sized portal zone with vein, artery and the bile duct branches (portal triad). Finally, Fig. 2f shows a region towards the metastasis center where scarce tumor, extensive necrosis, and fibrosis all appear within a 3 × 3 mm area. All main tissue components of the colorectal liver metastasis are observable in the x-ray as well as the classical histology. We tested 3 samples from three different patients, all showing a good visual agreement between x-ray and classical histology.

**Fig. 3:**
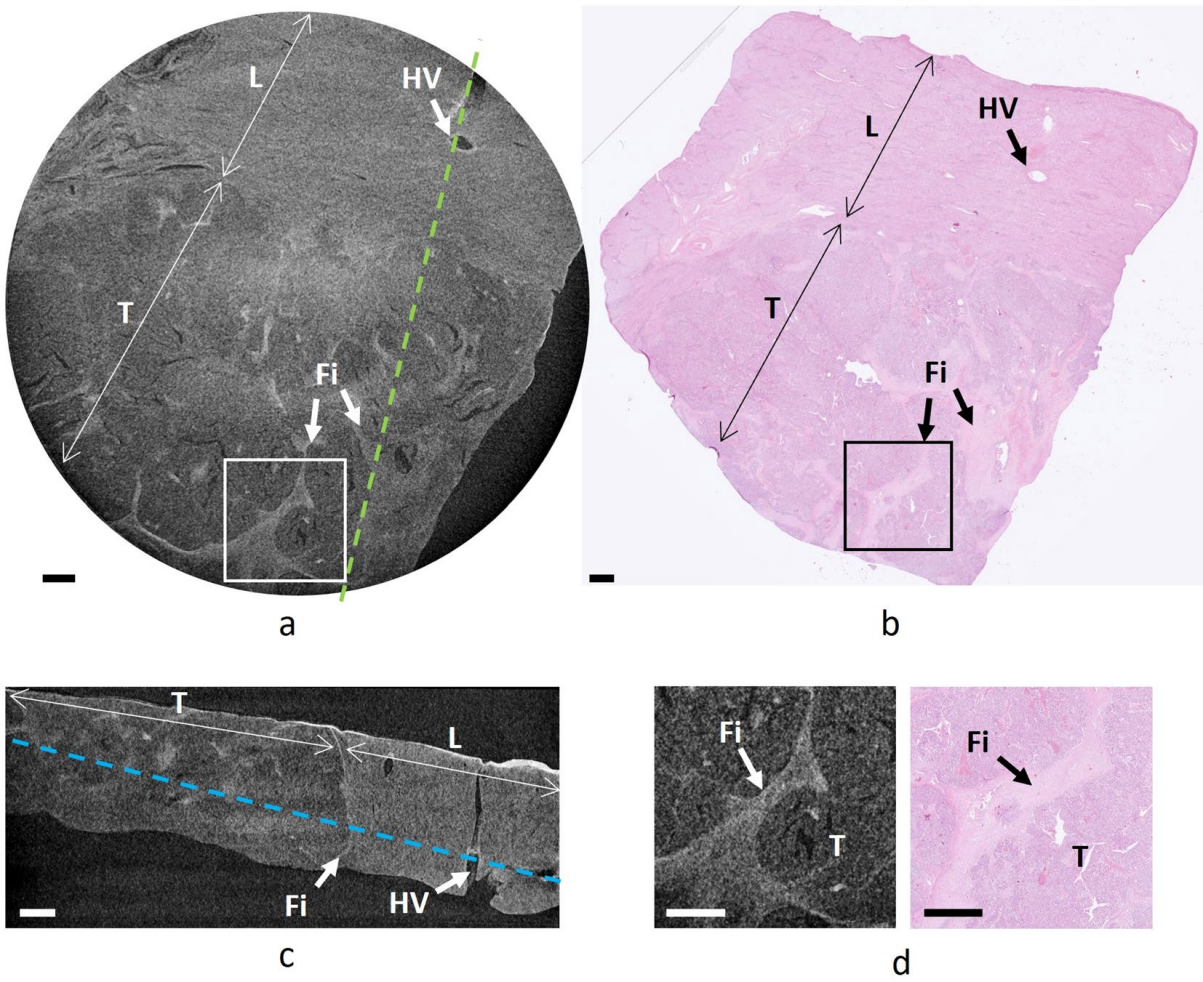
Hepatocellular carcinoma. (**a**) Virtual slice (MIP) through the microtomography volume, matching (**b**) the classical histology slice (H&E staining). The sample contains normal liver (L) and tumor (T). (**c**) Virtual slice through the microtomography volume, corresponding to the green dashed line in (**a**). The blue dashed line marks the position of the slice in (**a**). Fibrous septa (Fi) within and encapsulating the tumor and the hepatic vein (HV) can be seen. (**d**) Close-up of tumor (T) with intervening fibrotic septa (Fi) in x-ray (left) and classical histology (right). Scale bars: 1 mm.

Figure 3 compares the x-ray histology and the classical histology for hepatocellular carcinoma. Primary liver and bile duct cancers are the 3rd most deadly cancer type^24^. Hepatocellular carcinoma, here investigated, accounts for 75% of the primary liver tumors.

Figure 3a,b shows a virtual slice of the phase-contrast x-ray microtomography (a) that matches the classical histology slice (b). Both normal liver as well as tumor tissue are easily distinguishable in both images. In addition, we observe intratumoral fibrotic septa, encapsulating fibrosis and a medium sized hepatic vein. Figure 3c shows a section perpendicular to (a), to illustrate the 3D capacity of the x-ray tomography. Here we also observe tumor tissue, normal liver tissue, fibrous septa and encapsulations, and even a continuous longitudinal section along a hepatic vein. Finally, Fig. 3d shows a 4 × 4 mm close-up region of the tumor center with the septal fibrous pattern clearly observable in both modalities. Again, we tested 3 samples of hepatocellular carcinoma from three different patients with comparable results.

### Pancreas tumors

Here we show the results for the most common types of pancreatic cancer: Pancreatic ductal adenocarcinoma (n = 1) and the rare pancreatic neuroendocrine tumor (n = 1).

**Fig. 4:**
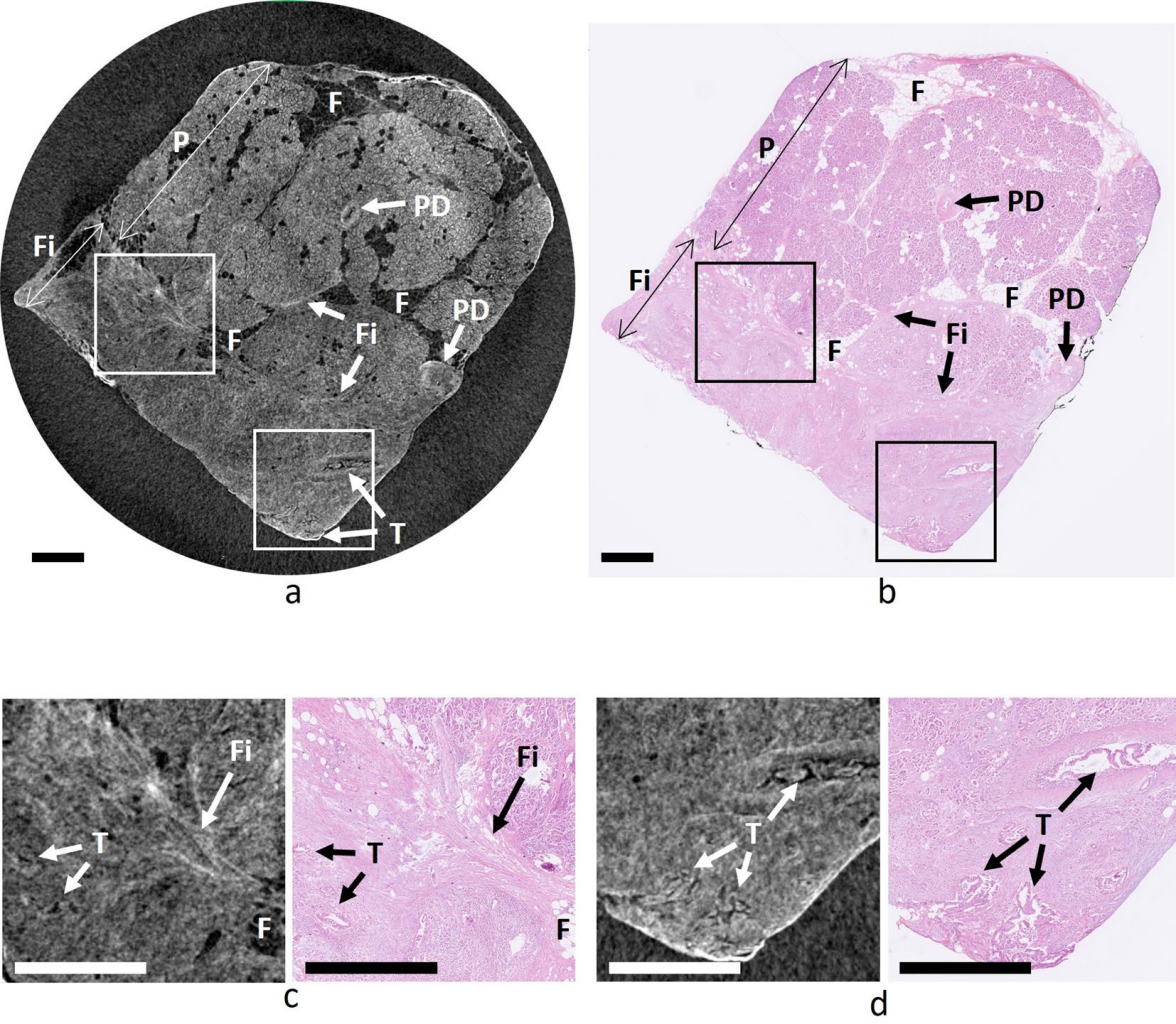
Pancreatic ductal adenocarcinoma. (**a**) Virtual slice through the microtomography volume, matching (**b**) the classical histology slice (H&E staining). The sample contains normal pancreatic tissue (P) and a region of intermixed fibrosis (Fi) and tumor. Pancreatic ducts (PD) and interlobular fat (F) can also be seen. (**c**) Desmoplastic fibrosis in micro-CT (left) and classical histology (right). (**d**) Large, irregular tumor glands (T) in x-ray (left) and classical histology (right). Scale bars 1 mm.

Figure 4 compares the x-ray histology and the classical histology for the pancreatic ductal adenocarcinoma. This cancer type accounts for 90% of the pancreatic cancers. It has a poor prognosis, with a five-year survival rate of 9% worldwide^25^.

Figure 4a,b shows a virtual slice of the phase-contrast x-ray microtomography (a) that matches the classical histology slice (b). We observe normal pancreatic as well as tumor tissue. In addition, fibrotic areas and ensembles of individual fat cells are visible, as well as the pancreatic ducts. Figure 4c shows that the desmoplastic fibrosis in which the tumor tissue is embedded is clearly visible also in the x-ray tomography. Finally, in Fig. 4d we observe infiltrating, large, irregular tumor glands near the edge of the tissue sample.

**Fig. 5:**
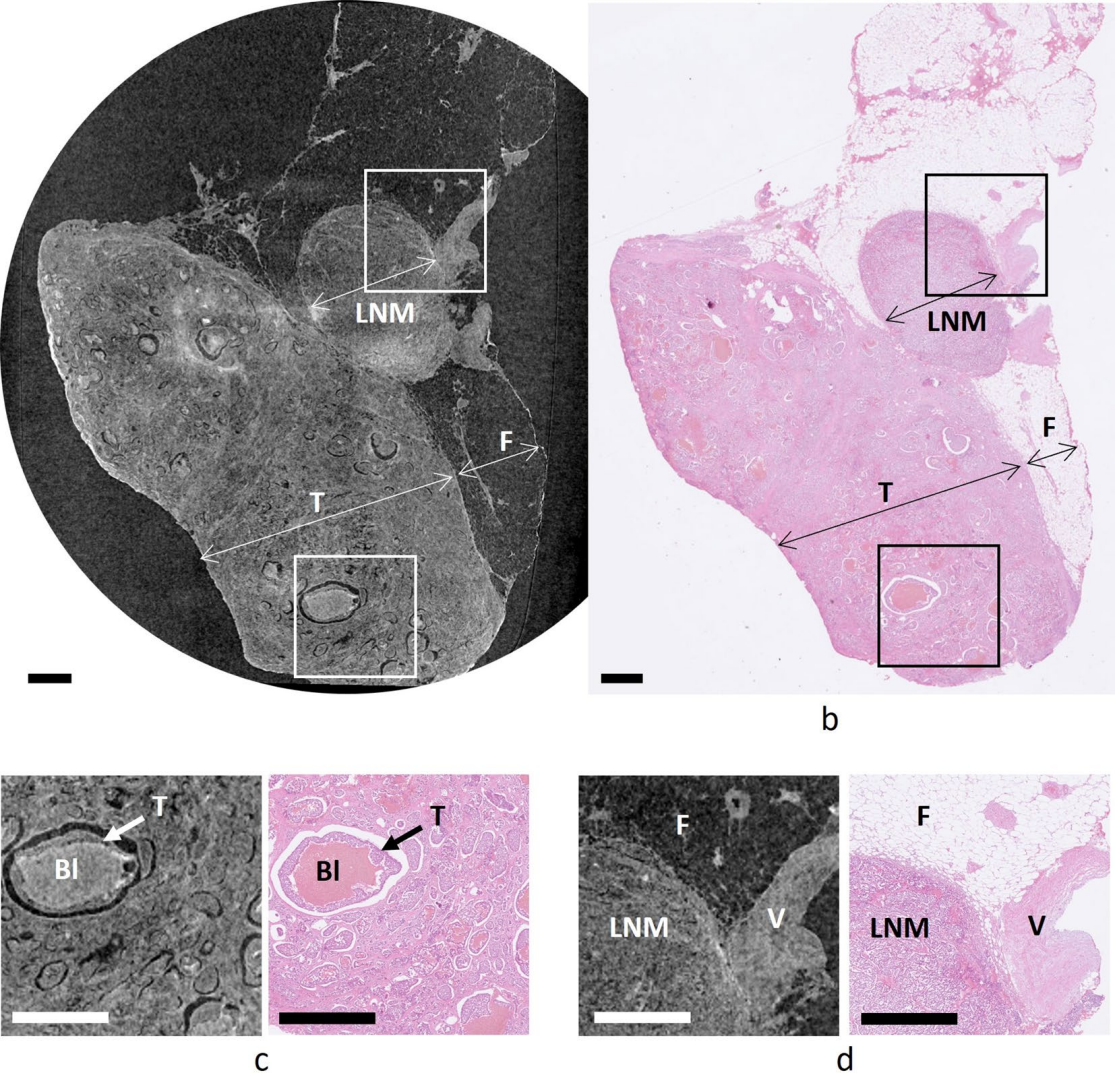
Pancreatic neuroendocrine tumor. (**a**) Virtual slice through the microtomography volume, matching (**b**) the classical histology slice (H&E staining). The sample contains peripancreatic fat (F), neuroendocrine tumor (T) and a lymph node metastasis (LNM). (**c**) Close-up of the tumor (T), in ring-like configuration, with blood (Bl). (**d**) Lymph node metastasis (LNM), fat (F) and vein (V) in the peripancreatic soft tissue. Scale bars: 1 mm.

Figure 5 compares the x-ray histology and the classical histology for the pancreatic neuroendocrine tumor. This tumor type is less common and accounts for 1–2% of the pancreatic cancers^26^.

Figure 5a,b shows a virtual slice of the phase-contrast x-ray microtomography (a) that matches the classical histology slice (b). We observe fat tissue, tumor tissue, and a lymph node metastasis, all clearly delineated. Figure 5c shows at high magnification a microscopic area where the tumor cells are configurated in a ring-like structure filled with blood. Finally, Fig. 5d shows a close-up of the lymph node metastasis in the peripancreatic soft tissue.

### Sarcomas

Here we show the results from two different sarcomas. Sarcoma is a rare, aggressive and heterogeneous group of tumors that arise throughout the body. The incidence is approximately 10% of pediatric cancers and 1% of all adult malignancies^27^. The value of resection margin for local control of the disease is well established for this group of cancers^28,29^^.^ However, their challenging growth pattern makes resection margin assessment difficult, pointing at the present limitations of our technique. A chondrosarcoma is presented in Supplementary Material (Fig. S7).

**Fig. 6:**
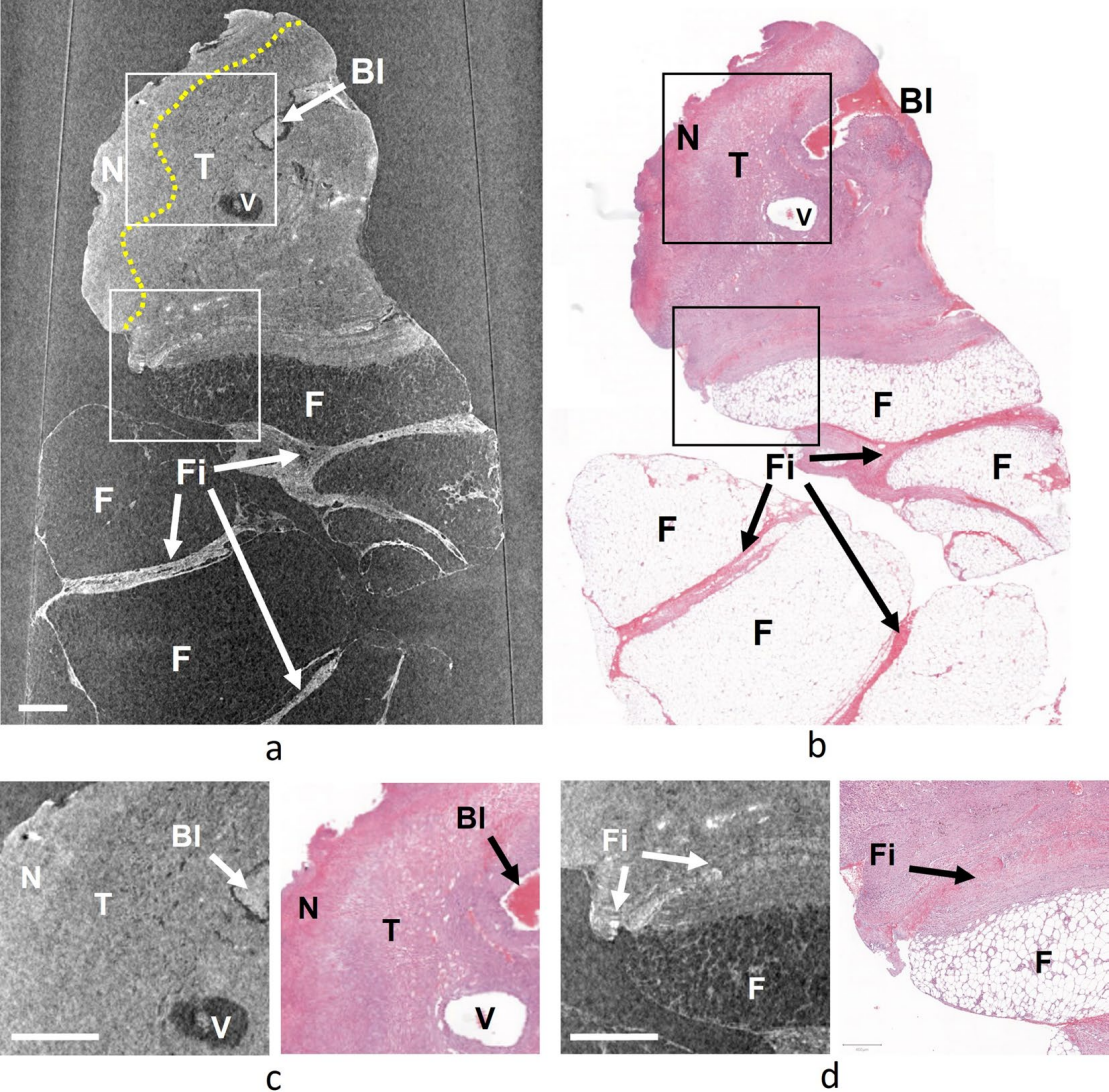
Osteosarcoma. (**a**) Virtual slice from the microtomography of a tumor biopsy matching (**b**) the H&E-stained section. The images show tumor tissue (T), a region of necrosis (N) delineated in yellow , blood (Bl) a blood vessel (V) and fat (F) with hemorrhagic fibrous septa (Fi). The lower panel shows magnified images in x-ray histology (left) and classical histology (right) of (**c**) the tumor-necrosis boundary. (**d**) Hemorrhagic connective fibrous tissue (Fi) between the tumor and the adjacent fat tissue. Scale bars: 1 mm.

Figure 6 compares the x-ray histology (a) and the classical histology (b) for an osteosarcoma. The phase-contrast image differentiates viable tumor tissue adjacent to an area of necrosis and healthy fat tissue. Collagen-rich fibrous tissue, blood, and a blood vessel can also be distinguished. Closer histopathological examination of the H&E section at a higher magnification confirmed the boundaries between tumor and necrotic tissue (c) and the presence of collagen fibers between the tumor and adjacent fat tissue (d).

**Fig. 7:**
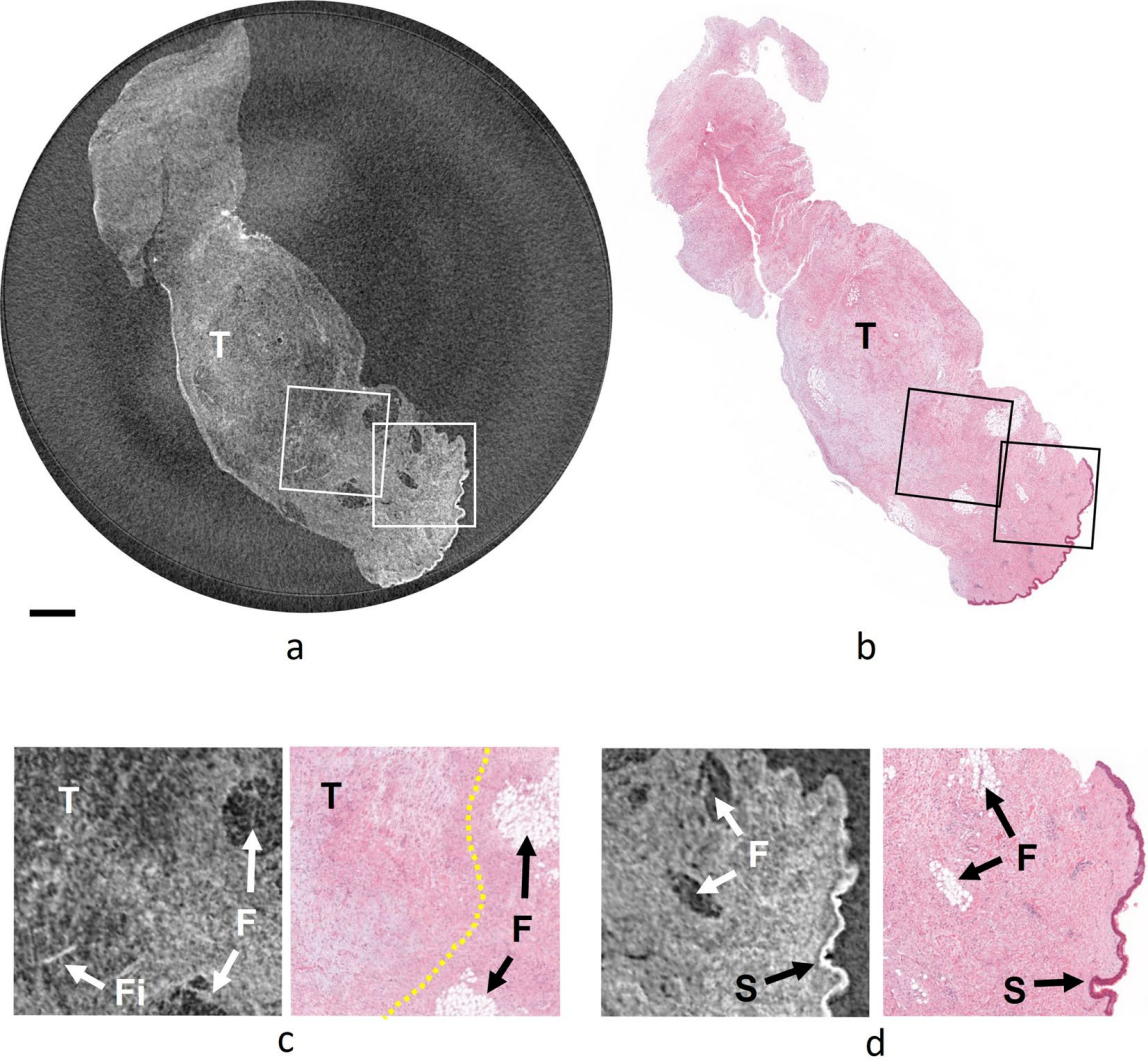
Low-grade fibromyxoid sarcoma. (**a**) Virtual slice from the x-ray microtomography of a tumor biopsy matching (**b**) the H&E-stained histology section. The tumor is characterized by a bland spindle cell population within an alternating fibrotic and myxoid stroma, with poorly circumscribed borders. The lower panels show the x-ray histology and the classical histology of (**c**) a closeup of the tumor border (yellow dashed line) with collagen-rich fibrosis (Fi) visible in the x-ray histology image and (**d**) overlying skin (S) and fat lobules (F). Scale bars: 1 mm.

Figure 7 compares the x-ray histology (a) and the classical histology (b) for a low-grade fibromyxoid sarcoma, a type of soft-tissue malignancy. The tumor is characterized by a bland spindle cell population within an alternating fibrotic and myxoid stroma. In Fig. 7c, a close-up of the tumor border is shown, visible in both the x-ray and the classical histology. Figure 7d shows a non-tumorous, overlying region of fibroadipose tissue and skin.

## Discussion

We have shown that phase-contrast x-ray microtomography of rapidly fixed and dehydrated tissue specimens has significant potential for 3D resection margin assessment. The method is demonstrated for several common tumor types in liver and pancreas as well as for rare tumor types such as sarcomas. For the upper abdominal cancers, it appears to provide reliable and repeatable results that can be interpreted by the clinical professional. For the sarcomas with their more complicated growth patterns, the assessment is less distinct and more work is needed to make it a standard tool.

The rapid fixation/dehydration is done with a single-step bath in concentrated acetone. This significantly increases the contrast in the phase-contrast x-ray tomography. The probable reason is differential shrinking between different types of tissue, the most important between tumor tissue and healthy tissue. Naturally, the degree of shrinking varies with, e.g., water content, amount of connective tissue etc. This explains the difference in signal between the different tumor types investigated. Naturally, depending on tissue type, morphology may be slightly more affected with our method than with the classical histology preparation using gradually increasing concentrations of, e.g., ethanol. However, for the purpose here, i.e., resection margin assessment, this is not a constraint.

Although it is not possible to quantify shrinkage and morphological changes within the same tissue samples we have compared acetone-fixed samples with adjacent tissue samples fixed in parallel with formalin only. Figures S8–S12 in the Supplementary Material shows that the morphological changes associated with acetone fixation do not hinder classical histological assessment. For completeness we note that classical fixatives like formaldehyde do not provide sufficient differential shrinkage to yield observable x-ray contrast (cf. Figs. S12 and S13 in Supplementary Material).

We note that classical histopathology with H&E staining remains reliable on the same samples that have previously undergone the single-step rapid acetone treatment and X-ray microtomography. Thus, meaningful comparison between the new X-ray histology and classical histology can be performed on the same samples. In a future clinical implementation, where perioperative patient risk and discomfort needs to be weighed against possible additional treatment, this feature can be exploited so that the 3D x-ray histology and the classical histology can be combined on the same specimen for improved diagnostic accuracy.

Key to the method is the high-resolution and high-contrast imaging. Our propagation-based phase-contrast microtomography provides approximately 10 µm resolution. Furthermore, the phase contrast results in improved soft-tissue contrast, primarily around borders between different tissue types (edge enhancement). Thus, the method is particularly sensitive for structures on the cellular scale and therefore can provide margin assessment with great spatial detail, as shown in Figs. 2, 3, 4, 5, 6, 7. Furthermore, infiltrating tumor groups consisting of a few to several cells close to the edge of the tissue sample are observable, as demonstrated in Fig. 4d and in Ref.^19^. In the present paper we observe cellular detail in the fat tissue (e.g., Figs. 4 and 5) while we do not have sufficient contrast to observe individual tumor cells. However, the tumor edge definition is on the cellular level.

Detection of ensembles of few cells requires imaging with high signal-to-noise ratio to allow observation of the minute shades of gray. The signal to noise depends on the photon statistics and imaging artifacts. Photon statistics depend on the exposure time and are currently not a limitation. The imaging artifacts are presently larger by creating a non-uniform background that makes detection of small ensembles of tumor cells difficult. These artifacts are presently reduced by ring artifact reduction, de-striping, and denoising algorithms (cf. “Materials and methods”) and we expect further improvements of these algorithms.

For completeness, we compare our method based on phase retrieval with an absorption-style reconstruction without the phase retrieval on the same projection data. Figure S14 in the Supplementary Material shows the result. In brief, the absorption-style image contains high-resolution details stemming from the non-linear edge enhancement in the projections. However, these structures are difficult to interpret due to their non-linear origin. Furthermore, the phase-retrieved image has higher contrast, probably since the non-linear edge enhancement results in the inconsistent projections in the absorption-style reconstruction.

The detailed x-ray histological assessment in 3D demonstrated here suggests that the method may have a more general applicability, well beyond the cancers studied here. The method is clearly applicable both to large specimens as well as to small biopsies. The details visible in the virtual microtomographic sections of the skin (Fig. 7c) suggest that the method could be useful in melanoma surgery. Clearly, very common cancers like breast, prostate, and thyroid should be investigated.

Although single-cell observability is a valuable asset of the present phase-contrast x-ray microtomography system, the experienced pathologist can also draw conclusions from larger-scale patterns. The uniform appearance of the tumor-free tissue sharply contrasts with the larger variations on different size-scales of the tumor tissue. Furthermore, the fibrous tissue often surrounding the tumor is easily identifiable and helps delineate the tumor. Finally, observation of histological landmarks in combination with knowledge of typical growth patterns for specific cancers may also assist in the pathologists’ assessment of the x-ray histology. This may become especially important in the sarcoma images, where it is presently more difficult to unambiguously assess the tumor/healthy tissue boundaries.

The present imaging procedure takes 4–5 h. This is divided into x-ray microtomography data acquisition (2–3 h), image pre-processing (< 1 h) and tomographic reconstruction (< 1 h). Even when accounting for a few hours of sample preparation in acetone, the whole procedure of the x-ray histology is still 10 times faster than the classical histology. Due to the transportation necessary in the present experiment (cf. “Materials and methods”), the samples were imaged a few hours to 24 h after the surgery. The longer exposure to acetone did not seem to influence the results.

Finally, the question is whether the total time can be reduced by another factor ten to allow for intraoperative feedback to the surgeon. We believe this is possible. The time for the x-ray data acquisition can be reduced by a factor of 10 by exchanging the present Excillum 125 W D2 source for a > 1 kW MetalJet F source (Excillum AB), although this high-power source will need some modifications to allow single-cell observability. We also expect to reduce the tomographic reconstruction to 10 min by massive parallelization in the cloud. The acetone sample fixation time is expected to be reduced to 10–20 min, by forced perfusion. In total we hope to provide full 3D x-ray resection margin analysis within 1 h. This would allow intraoperative feedback and potentially large patient benefit.

Although promising, there are still significant additional steps that needs to be taken before the presented method can be translated to the clinic. Below we discuss a few.

The current workflow and presented data is derived entirely from standard histopathology cassettes-sized tissue samples, and not from entire surgical specimens. Although rapid acetone fixation and subsequent imaging are in our opinion adequate for a tissue level assessment of the tumor border based on x-ray, we are not yet at the point where the technique can be readily applicable to resection specimens. We can foresee a progressive technical development starting with smaller resection specimens, e.g., local resections of liver metastases, through mid-sized resections like thyreoidea and breast sectors, ending with larger resections like the hepatic and pancreatic ones. Along the path, fixation, imaging, x-ray system, processing, and interpretation will need to evolve, tailored to the different types of specimens and tissues and improved, e.g. by using perfusion systems for larger specimens.

Although one of the main potential for clinical application of this technology is resection margin assessment, in the current study we have rather focused on tumor and tissue delineation and expert consensus-based correlation between x-ray and pathology. To address this limitation, we are currently conducting with promising results a continuation retrospective study in which pathologists and radiologists blindly quantify the minimal distance from tumor to the resection margin in resection specimens of colorectal liver metastasis and hepatocellular carcinoma. This study will provide relevant data specifically on resection margin assessment in a quantitative way.As noted above, the present contrast is insufficient to reliably detect single cells and small cell nests in highly invasive tumors, e.g., PDAC, or their propagation in the form of perineural and lymphovascular spread. Further development and improvements will be required to achieve such observable cell-level resolution.

As regards architectural and resource planning, space in the operation theaters and the pathology departments is usually limited and already occupied by different types of equipment. Adding a new, additional one will require careful planning where closeness to the operation theater and the actual physical space available will need to be balanced.

Finally, assessment criteria will need to be defined and subject to clinical-grade diagnostic quality standards. As a novel diagnostic modality at the interface between radiology and pathology, it is not clear at this early point of technology development whether radiologists or pathologists will be primarily responsible for the actual diagnosis. Regardless, tailored training will be needed for the healthcare professionals that will both operate the equipment and assess the images.

## Materials and methods

### Ethics statement

The research was approved by the Swedish National Ethical Review Authority, permits #2019-00583 and #2022-01556-02 for liver and pancreas studies, and #2022-05409-01 and #2013-1979-31 with amendment #2018/1998-31-01 for the sarcoma studies. Informed consent was obtained for every participant patient. The participants received no compensation. All methods were performed in accordance with relevant institutional guidelines and regulations.

### Sample preparation

Fresh tissue samples, consisting of typically 5 mm thick slices, < 25 × 25 mm, were obtained immediately after surgery and dropped into acetone in a 12- or 25-mm diameter plastic vial at room-temperature. Acetone is a strong dehydrant with rapid penetration into tissue. The samples were kept in acetone during transportation from the hospital to the phase-contrast imaging laboratory at Royal Inst of Technology (KTH) and typically imaged same day or the day after surgery. Directly after imaging, the samples were returned to the hospital for subsequent formalin fixation and classical histology.

### Laboratory propagation-based phase-contrast imaging system.

The laboratory phase-contrast system is described in detail in Refs.^15,16,19^ and depicted in Fig. S1. It relies on a liquid–metal-jet microfocus x-ray source, a high-resolution scintillator-coupled x-ray camera, and the sample on a rotating stage. We use a MetalJet D2 (Excillum AB, Sweden) operated at 70 kV and 125 W, focused to a 10 × 40 μm spot on the Galinstan jet. The rotation stage was a Newport URS100BCC (Newport, California) and the detector a Photonic Science (Photonic Science Ltd, UK) CMOS detector with 4096 × 4096 pixels with a pitch of 9 μm, fiber-optically coupled (1:1) to a 10 μm gadolinium oxysulfide scintillator. The detector point-spread function had a full-width-half-maximum (FWHM) of 22 μm.

### Imaging parameters

The source-sample distance (*R*_1_) and the sample-detector distance (*R*_2_) define the geometry (cf. Fig. S1 in Supplementary Material). The magnification of the sample to the detector is *M* = (*R*_1_ + *R*_2_)/*R*_1_ enabling imaging at a resolution better than the detector pixel resolution. The phase contrast is achieved from an effective propagation distance *z*_*eff*_ = *R*_1_*R*_2_/(*R*_1_ + *R*_2_), due to the divergent cone beam^30^. *M* and *z*_*eff*_ can be set independently by changing *R*_1_ and *R*_2_, and were adjusted to obtain good phase contrast and signal-to-noise ratio^30^ while keeping as much as possible of the sample in the field of view. Contrast peaks at *z*_*eff*_ = 1/2λ*u*^2^, where λ is the wavelength and *u* are the spatial frequency. In the present study we optimized the system to allow for detection with cellular resolution, i.e., the 10-μm range, while still maintaining exposure times reasonable. Typical settings were* R*_1_ = 300 mm and *R*_2_ = 300–400 mm resulting in a field of view of 15–18 mm given the 40 × 40 mm detector. For larger samples, we can offset the center of rotation on the detector and scan 360 degrees for a 30–36 mm field of view. Typical exposure times in the present experiments were 2–3 h during which 3600 projections were acquired.

### Observability in phase-contrast x-ray imaging

In the present paper we rely on propagation-based phase-contrast imaging (PBI). PBI typically detects the transverse Laplacian of the phase (∇^2^ϕ) with a single measurement and without extra optical elements. Compared to other methods like grating-based imaging (GBI), PBI results in the shortest exposure times but phase retrieval is usually necessary for further analysis. For intraoperative use, exposure time and resolution are critical. There are only few comparisons on observable detail vs dose/exposure time for different methods but for imaging gas-filled structures (like CO_2_-filled blood vessels or air-filled lung alveoli) the necessary exposure time for observing sub-50-μm structures may differ a factor ten in favor of PBI^31^. The method is readily expandable to 3D imaging via tomographic methods^32^.

### Image analysis and algorithms

The raw projection images, which contain the sample absorption and the Laplacian of the phase shift, were first dark- and flat-field corrected, and then phase retrieved using Paganin’s method^33^, with δ/µ = 1 × 10^–8^ and λ = 0.124 nm. The phase-retrieved images were tomographically reconstructed using the FDK cone-beam algorithm from Octopus Reconstruction (TESCAN, Gent, Belgium). Multiple algorithms for ring artifact reduction where employed, most importantly compensation for inhomogeneities in the scintillator thickness, but also de-striping of the sinograms and de-noising^34–38^. After finding the virtual slices in the reconstructed volume corresponding the classical histology slices, maximum intensity projections (MIP) over 50 µm thick slabs were performed for each virtual slice of the liver samples to enhance visibility (Dragonfly, Comet Technologies Canada Inc). The x-ray images from pancreas and sarcoma are shown as single virtual slices. Grey scales have been adjusted in all images. ImageJ (National Institute of Health, Bethesda, Maryland) and Dragonfly were used for post-processing and volume renderings.

### Comparative histopathology

After x-ray imaging, the tissue sample was returned to the pathology laboratory and processed for standard histology using paraffin-embedding, sectioning close to the surface of the tissue block after a short initial trimming to achieve a complete and even cut plane, and H&E staining. The histology slide was thereafter scanned at 40× magnification using av Hamamatsu Nanozoomer S360 slide scanner (liver and pancreas) and a Zeiss Axioscan 7 system with a QuPath imaging analysis (sarcomas). The whole slide image (WSI) was assessed by a pathologist (CFM, EP) specialized in the field who identified the main tissue types (e.g., tumor, non-tumorous), tissue components (e.g., fibrosis, necrosis) and tissue landmarks (e.g., porta zones, central veins) in the histology section. X-ray imaging experts (JR, HMH) independently identified the slab in the tomographic stack closest to the standard histology WSI and assessed the observable structures there at microscopical scale. Finally, pathologists and x-ray imaging experts identified and compared by consensus visual assessment the tissue types, components, and landmarks between both image modalities.

### Comparative fixation protocols

In the comparison with classical fixation schemes in the Supplementary Material (Figs. S12 and S13), the samples were immersed in formalin and acetone for one to a few days before the x-ray imaging. After that they were processed for standard histology (cf. above) before H&E staining.

## Supplementary Information

Supplementary Information.

## Data Availability

Full 3D stacks for Figs. 2, 3, 4, 5 is available on X-ray phase-contrast microtomography of acetone-fixed tumor tissue (https://datarepository.kth.se/records/kgb70-5ps61). All other data are available on request.
