# Supplementary material for: X-ray histology on rapidly fixed fresh tumor tissue samples for fast resection margin assessment
Source: Sci Rep. 2026 Jul 11;16:21642. doi: 10.1038/s41598-026-61069-6 (PMC13354771; doi:10.1038/s41598-026-61069-6)

SUPPLEMENTARY MATERIAL

X-ray histology on rapidly fixed fresh tumor tissue samples for fast resection margin assessment

Jenny Romell[1,2+], Carlos Fernandez Moro[3,4+], Bertha Brodin[1], Laszlo Szekely[3,4], Edvin Porovic[3], Panagiotis Tsagkozis[5], Ernesto Sparrelid[6], Mikael Björnstedt[3,4], and Hans M. Hertz[1*]

[1]Dept. of Applied Physics, KTH Royal Inst of Technology/Albanova, SE-10691 Stockholm, Sweden

[2]Excillum AB, Torsnästorget 17, SE-164 40 Kista, Sweden

[3]Department of Clinical Pathology and Cancer Diagnostics, F46 Karolinska University Hospital, Stockholm, SE-141 86, Sweden

[4]Department of Laboratory Medicine, Division of Pathology, Karolinska Institute, Stockholm, Sweden.

[5]Department of Acute and Reparative Medicine, Karolinska University Hospital, Stockholm, Sweden.

[6]Division of Surgery and Oncology, Department of Clinical Science, Intervention and Technology, Karolinska Institute, Karolinska University Hospital, Stockholm, Sweden.

[+] contributed equally

* E-mail: hertz@biox.kth.se

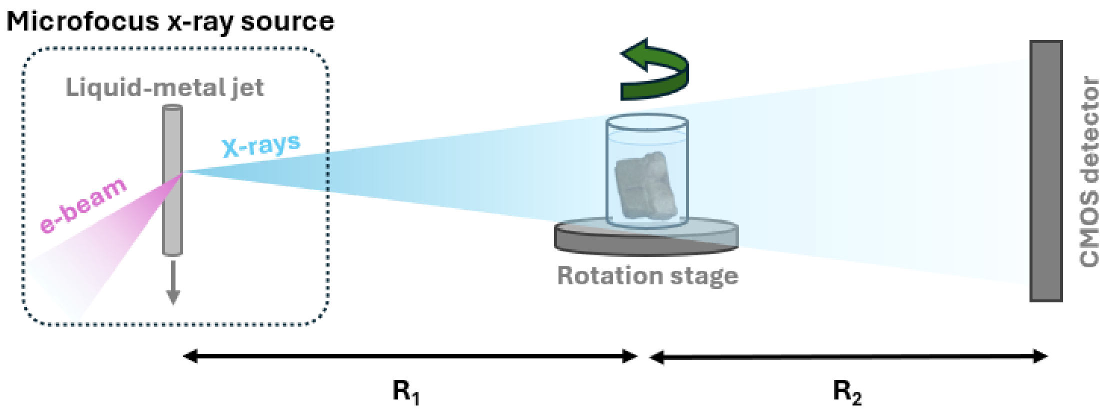

Figure S1. Imaging arrangement. The imaging distances R_1 (source to sample) and R_2 (sample to detector) define the geometric magnification M and effective phase propagation distance z_{eff}.

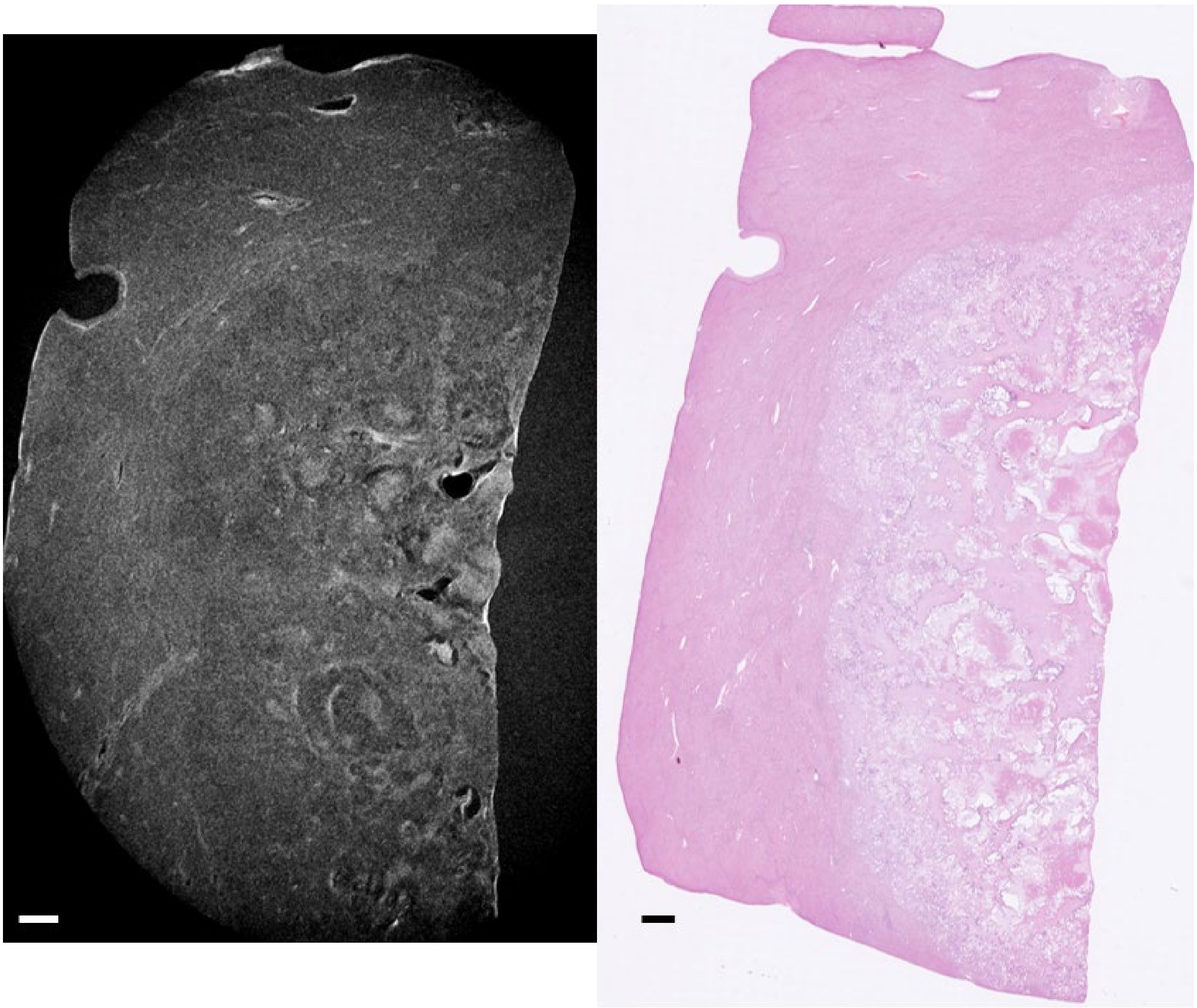

Figure S2. Colorectal liver metastasis. Phase-contrast CT (left) and matching classical histology slice (right). Scalebars: 1 mm.

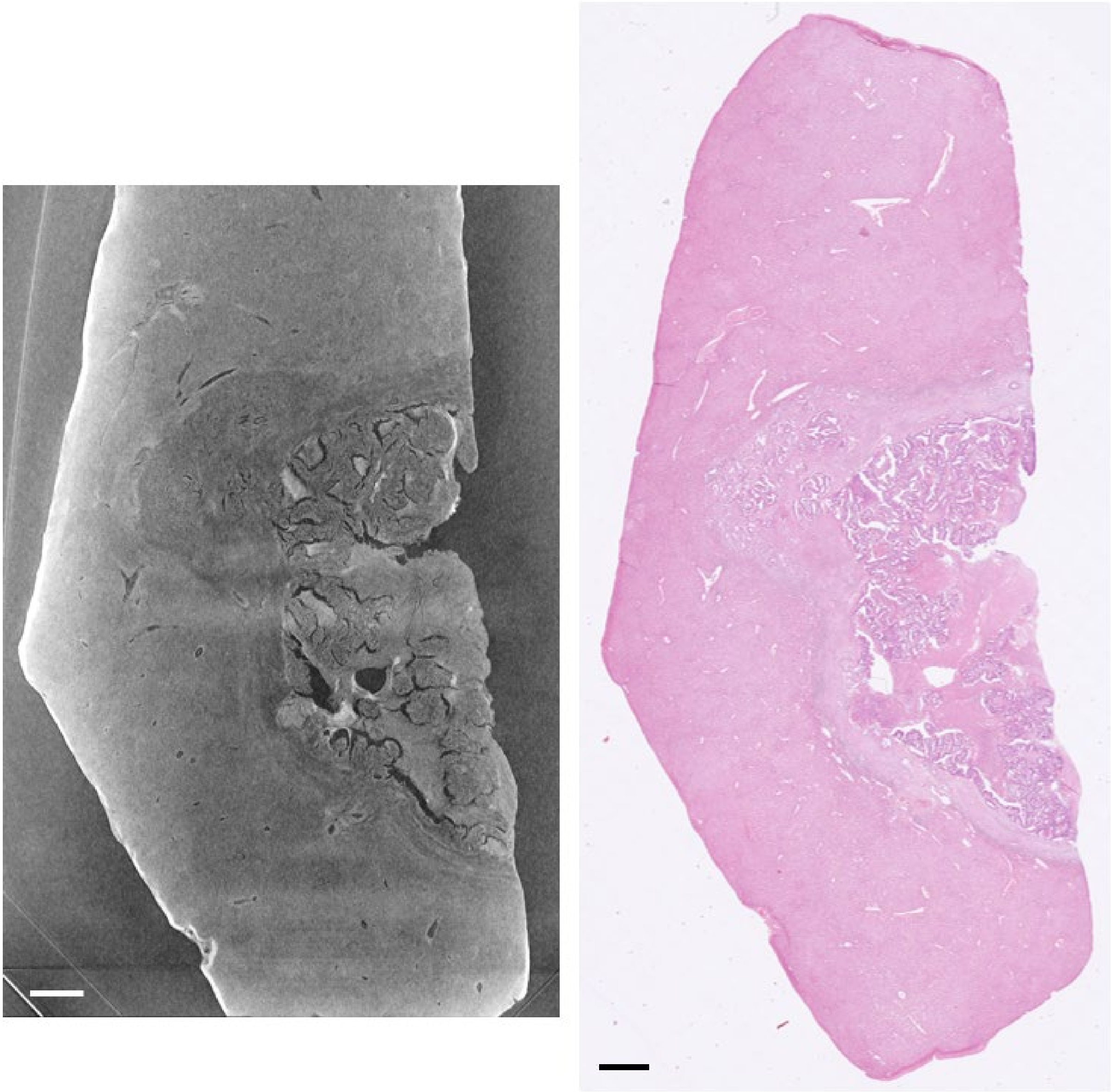

Figure S3. Colorectal liver metastasis. Phase-contrast CT (left) and matching classical histology slice (right). Scalebars: 1 mm.

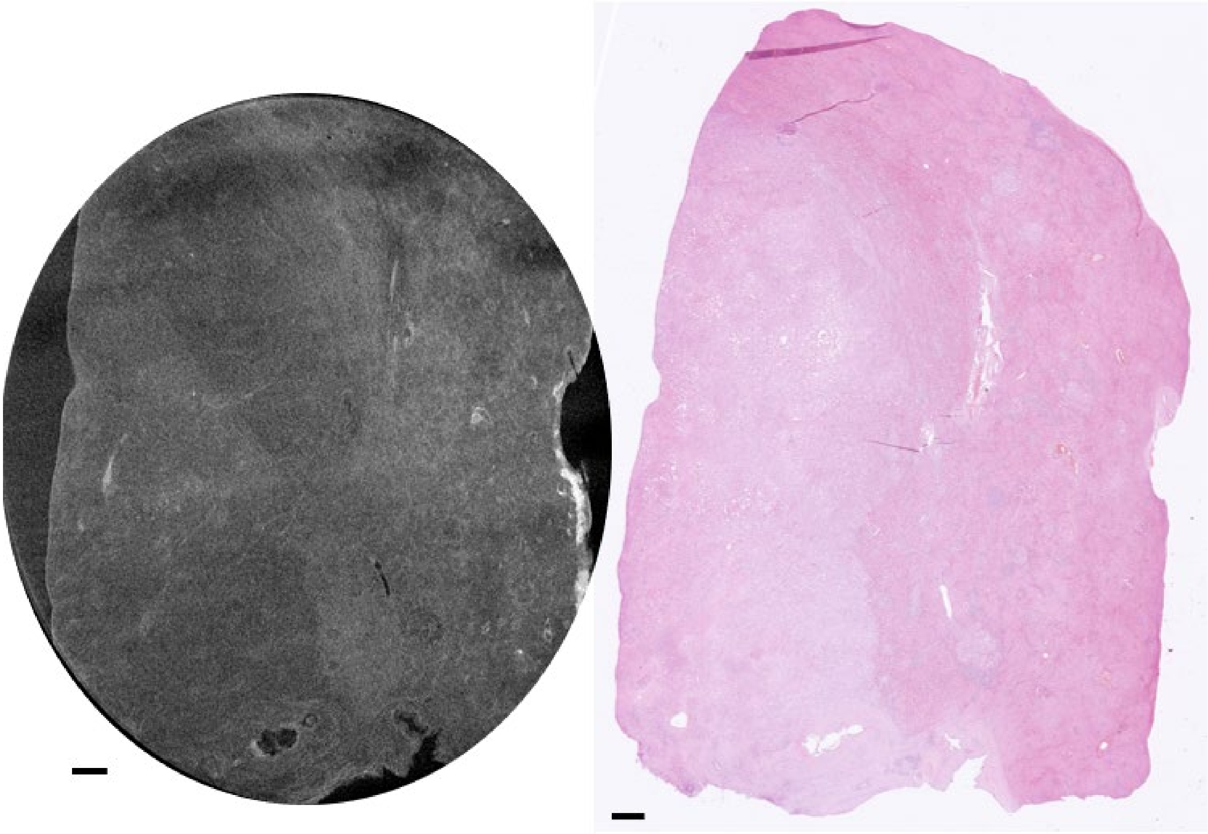

Figure S4. Cholangiocarcinoma. Phase-contrast CT (left) and matching classical histology slice (right). Scalebars: 1 mm.

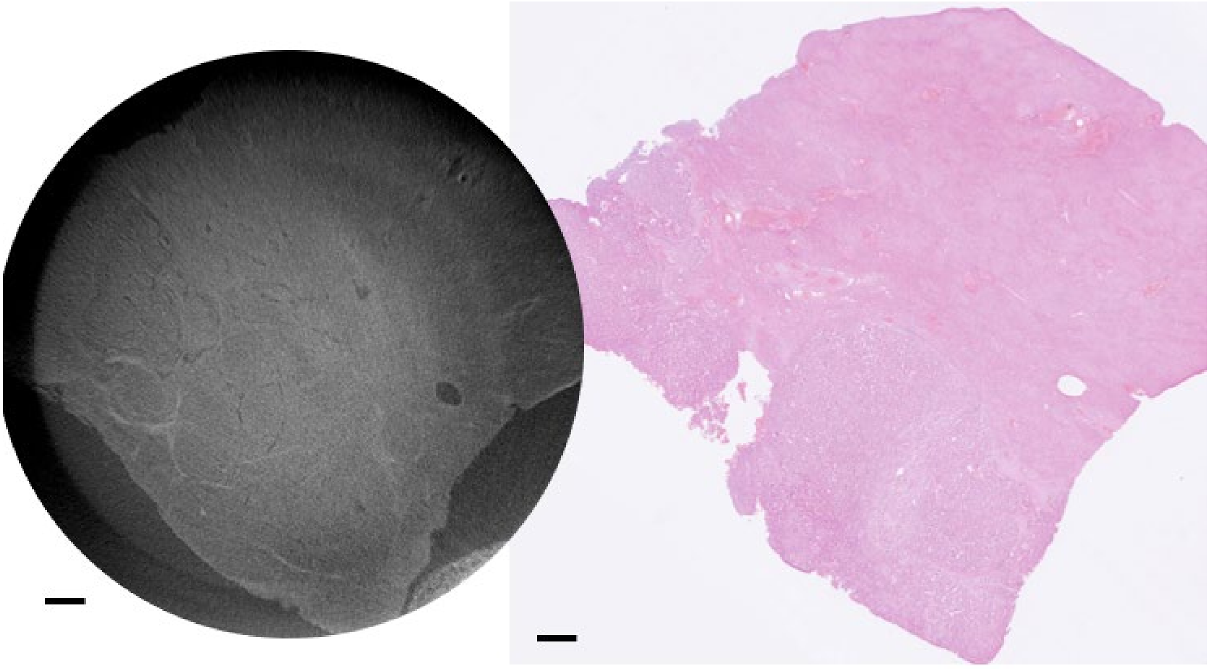

Figure S5. Hepatocellular carcinoma. Phase-contrast CT (left) and matching classical histology slice (right). Scalebars: 1 mm.

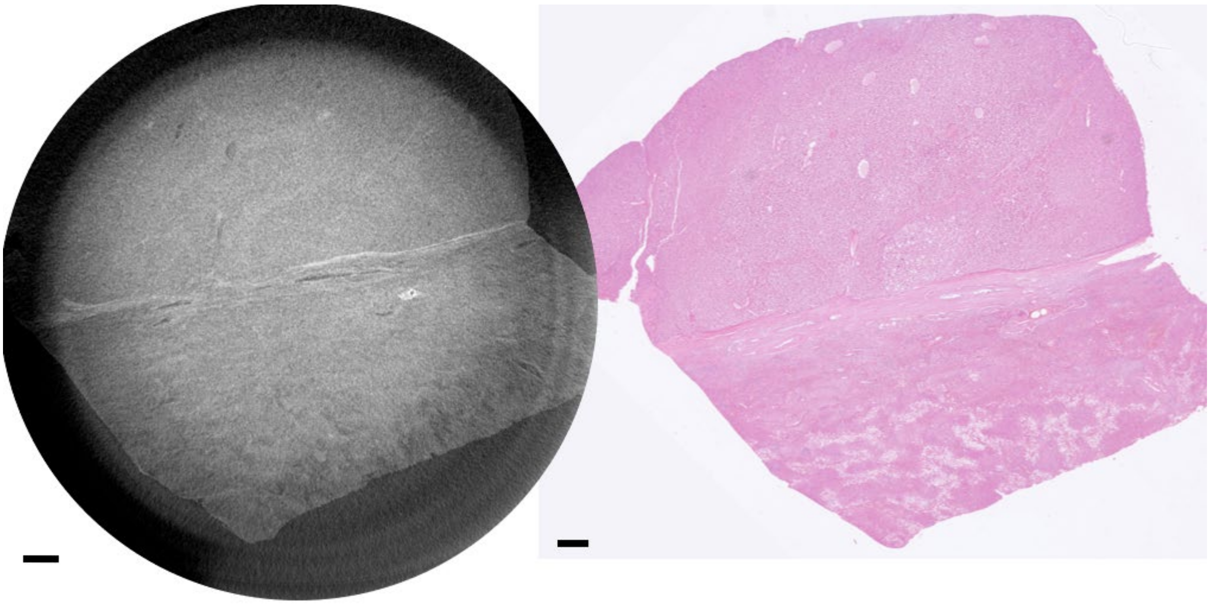

Figure S6. Hepatocellular carcinoma. Phase-contrast CT (left) and matching classical histology slice (right). Scalebars: 1 mm.

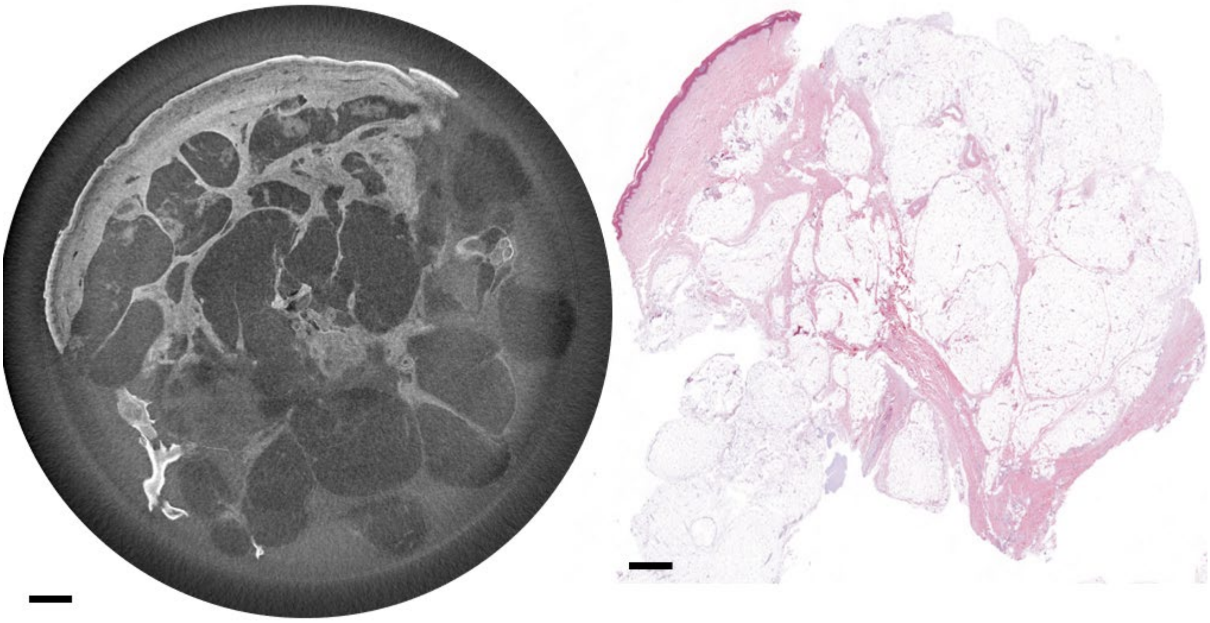

Figure S7. Chondrosarcoma. Phase-contrast CT (left) and classical histology slice (right). Scalebars: 1 mm.

Shrinkage I:

Quantitative measurements on pork tenderloin indicate a shrinkage due to acetone treatment of 5-15% (F. Lagerqvist, Degree Project, KTH Royal Inst of Technol. (2022); diva2:1686451).

Shrinkage II:

Representative photomicrographs of hematoxylin-eosin stained surgical specimen comparing matched sections fixed in acetone (for rapid pre-imaging fixation, followed by post-fixation in formalin after x-ray imaging) with comparable section fixed in formalin alone.

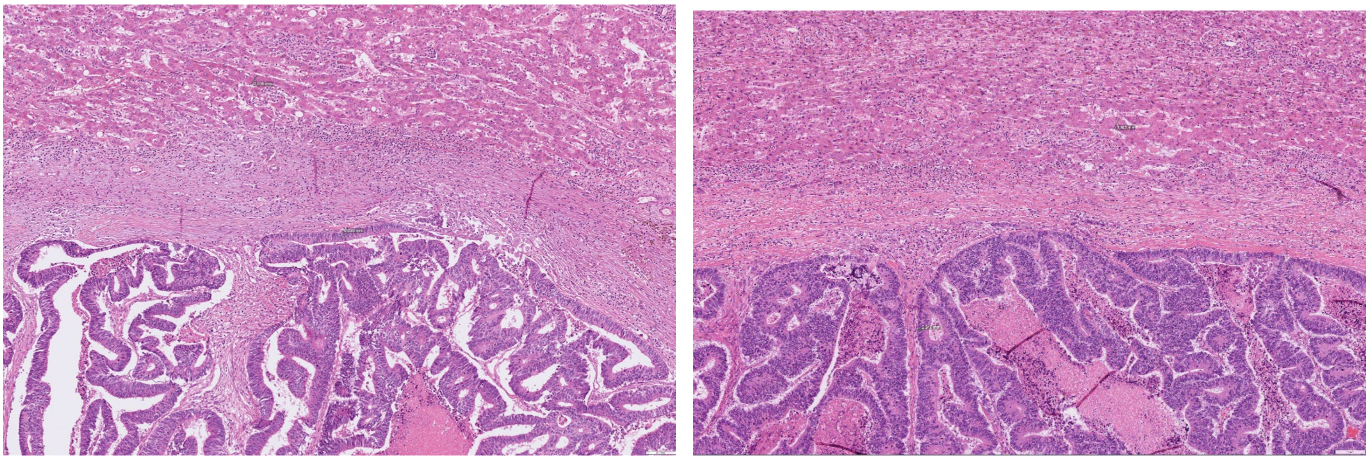

Acetone Formalin

Fig. S8. Colorectal liver metastasis: Tumor to the bottom, liver parenchyma on top, a fibrotic rim separating them (encapsulated growth pattern). Colorectal cancer cells show on visual assessment minimal shrinkage in acetone fixation. Measurements taken for tumor cell cytoplasm height: 36 µm acetone, 40 µm formalin; and liver cell plate thickness: 36 µm acetone, 40 µm formalin. Scale bars, 100 µm.

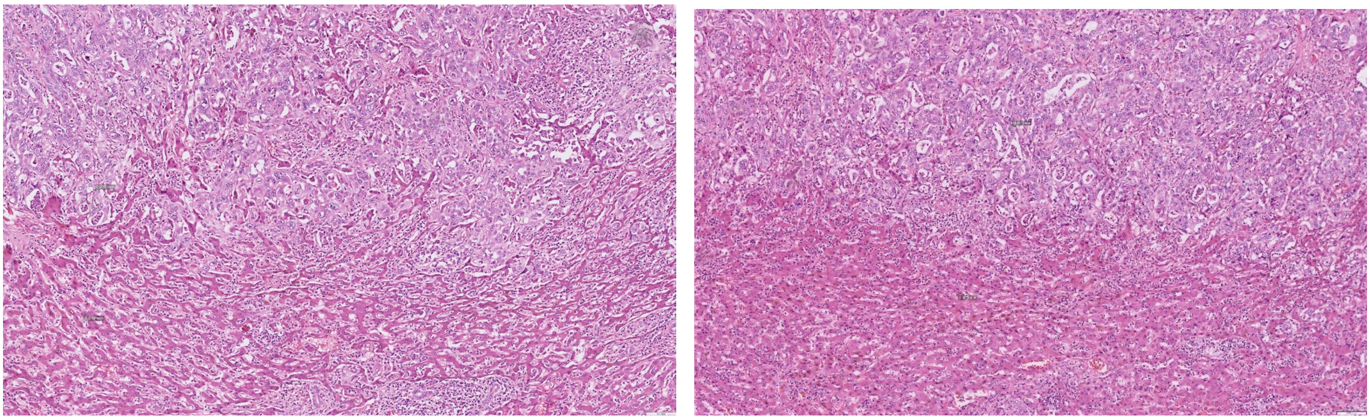

Acetone Formalin

Fig. S9. Intrahepatic cholangiocarcinoma: Poorly differentiated tumor on top, liver parenchyma to the bottom, the tumor cells invade surrounding liver cell plates that are flattened (replacement type 2 growth pattern). Cancer cells show on visual assessment minimal shrinkage in acetone fixation. Measurements taken for tumor cell cytoplasm height: 20 µm acetone, 22 µm formalin; and liver cell plate thickness: 19 µm acetone, 21 µm formalin. Scale bars, 100 µm.

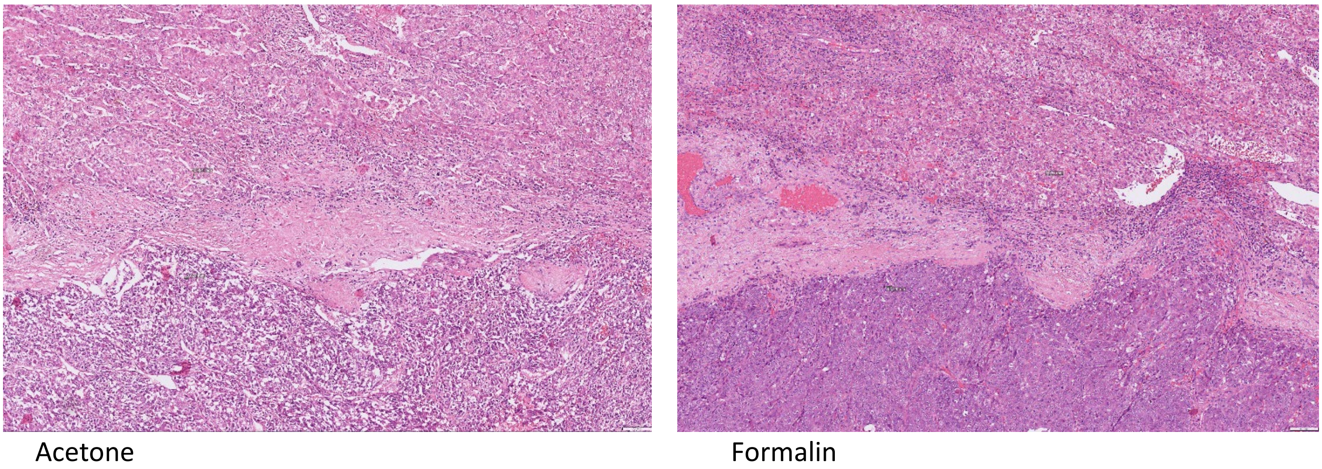

Fig. S10. Hepatocellular carcinoma: Poorly differentiated tumor to the bottom, liver parenchyma on top, a fibrotic rim separating them (encapsulated growth pattern). Hepatocellular carcinoma cells in this poorly differentiated tumor show moderate shrinkage in acetone fixation, giving rise to intercellular spaces, but the cancer cells are morphologically distinguishable as well as their relation to adjacent tissues. Measurements taken for tumor cell cytoplasm : 21 µm acetone, 24 µm formalin; and liver cell plate thickness: 31 µm acetone, 35 µm formalin. Scale bars, 100 µm.

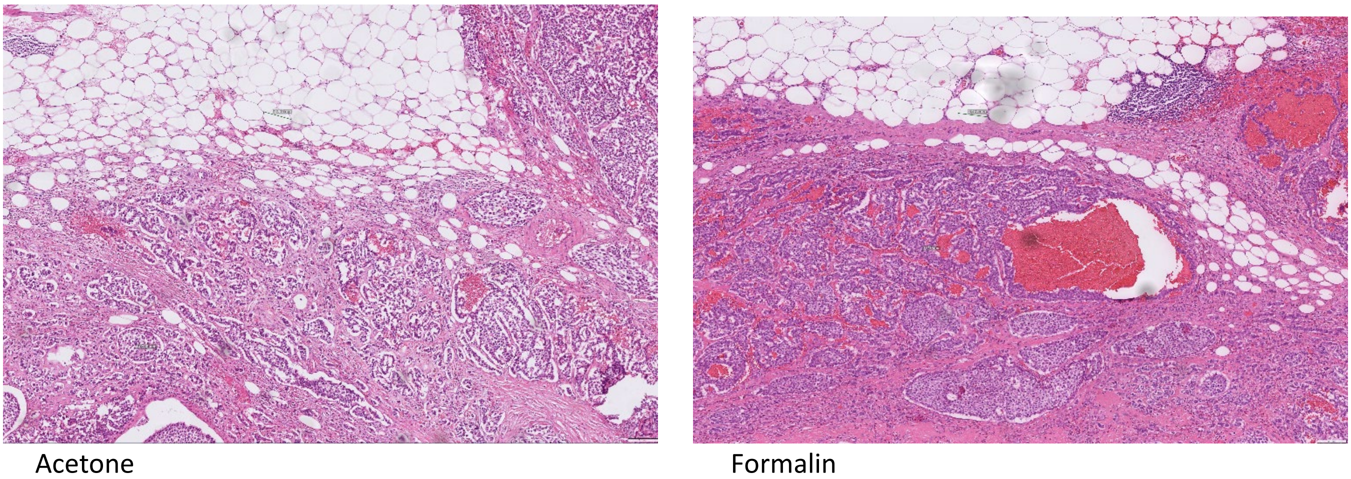

Fig. S11. Pancreatic neuroendocrine tumor: Tumor to the bottom, peripancreatic peripancreatic fat tissue on top. Cells in this well differentiated neuroendocrine tumor show mild shrinkage in acetone fixation, giving rise to discrete retraction artefact, but the cancer cells are morphologically distinguishable as well as their relation to adjacent tissues. Measurements taken for tumor cell nucleus : 8 µm acetone, 10 µm formalin; and adipocyte diameter: 97 µm acetone, 102 µm formalin. Scale bars, 100 µm.

Contrast comparison

Figure S12. Contrast comparison in x-ray imaging between acetone- and formalin-fixed healthy tissue from liver (space bar 1 mm). Below we show classical histology of the same samples (space bar 2.5 mm, rotated)

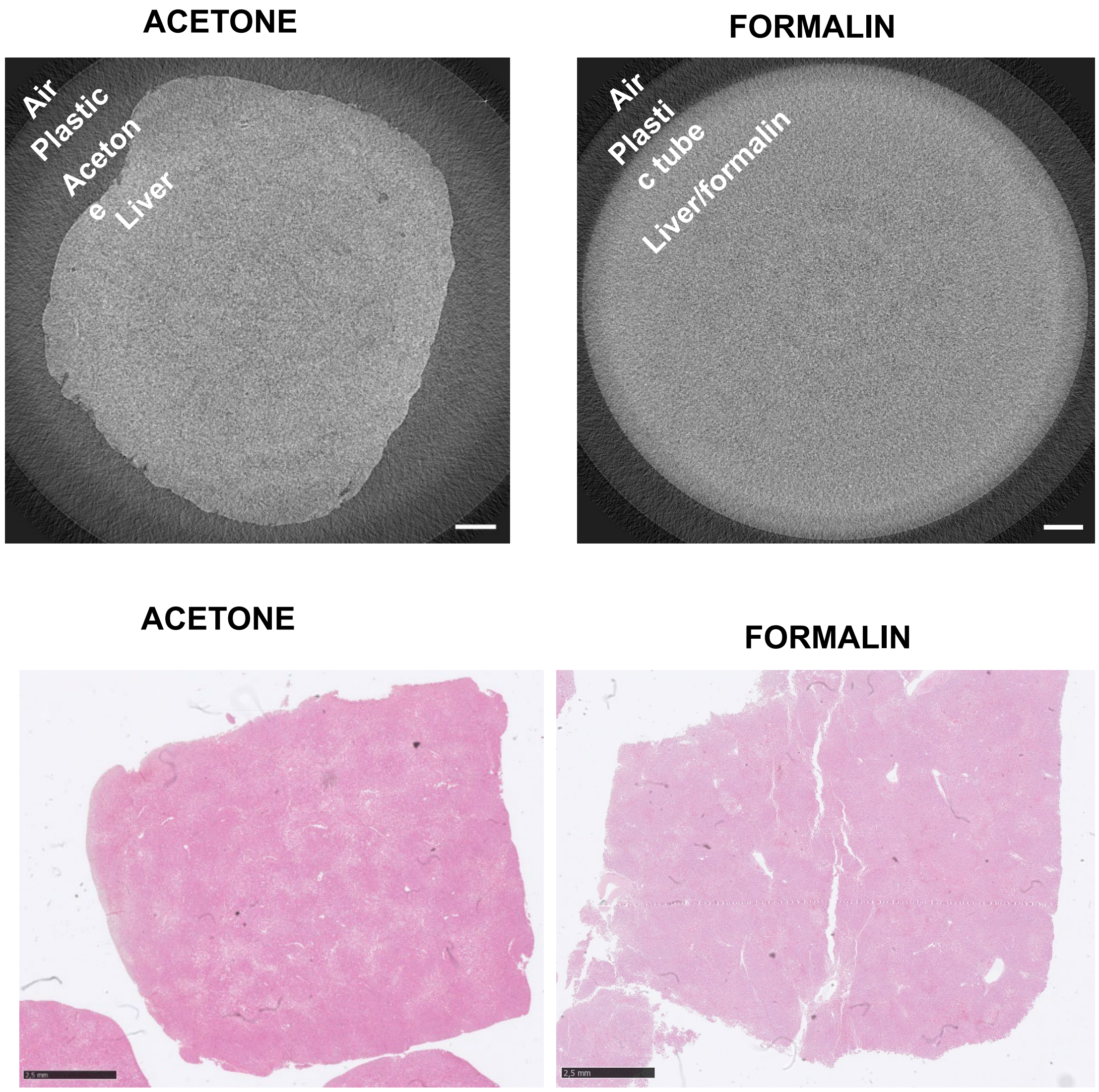

Figure S13. Contrast comparison in x-ray imaging between acetone- and formalin-fixed healthy tissue from pancreas (space bar 1 mm). Below we show classical histology of the same samples (space bar 2.5 mm, rotated))

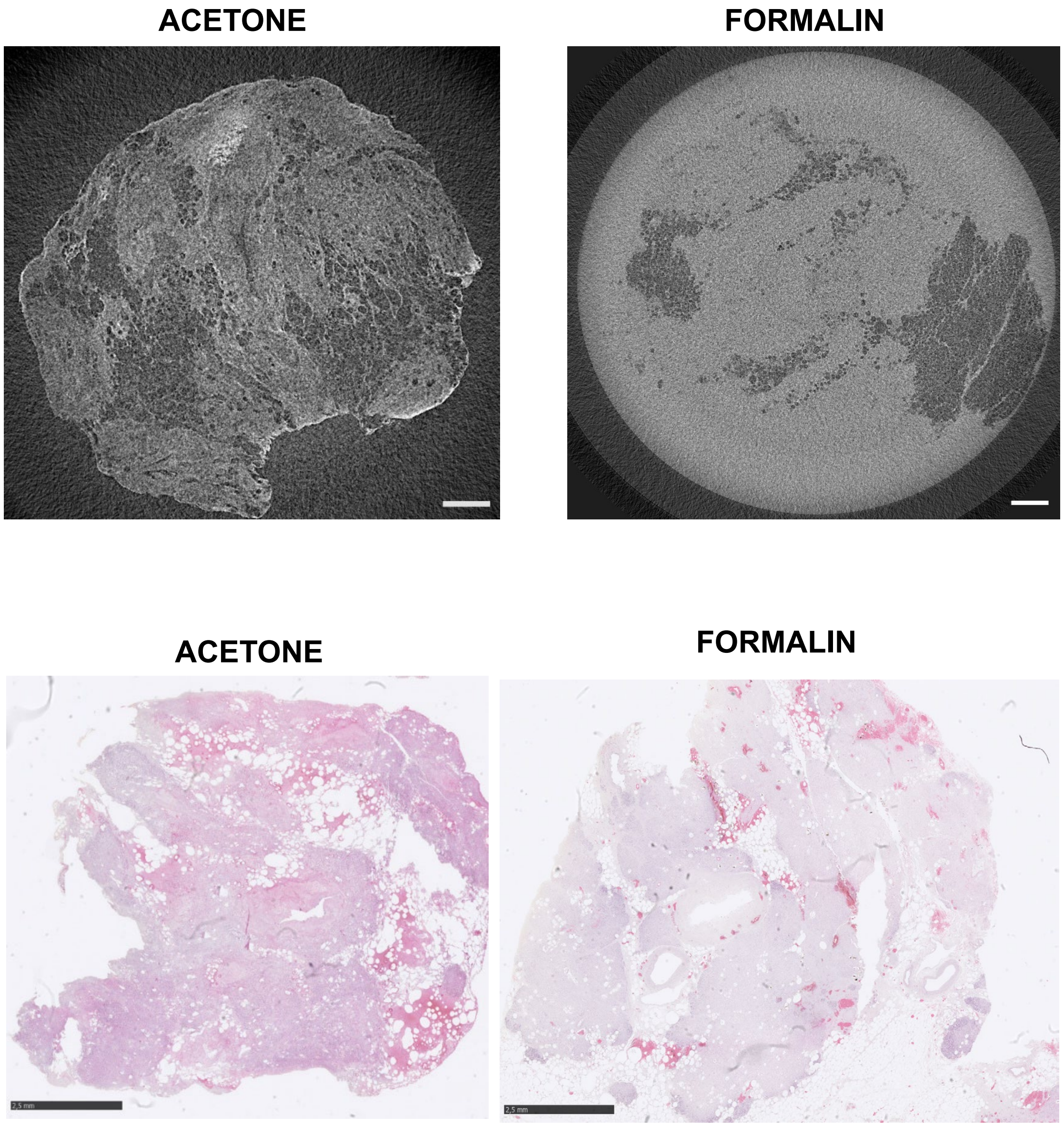

Comparative reconstruction methods – the importance of phase retrieval

Here we compare reconstructing our data without phase retrieval (as if it was absorption data) with the reconstruction of the same data with phase retrieval (i.e,, what is done in this paper). All other processing, e.g., background corrections, image artifact reduction etc, are the same.

Figure S14: Using the projection data of Fig. 2 in the MS (colorectal liver metastasis), the 3D image was reconstructed with and without phase retrieval. Left: Without phase retrieval. Right: With phase retrieval.

We observe that the phase-contrast image shows higher and more uniform contrast. In the absorption-style image the non-linear edge enhancement results in high-resolution structures of similar size as in the phase-contrast image. However, these are difficult to interpret due to their non-linear origin, which also results in a the loss of contrast. Note that the image in Fig. 2 in the MS were sliced to correspond with the actual classical histology.

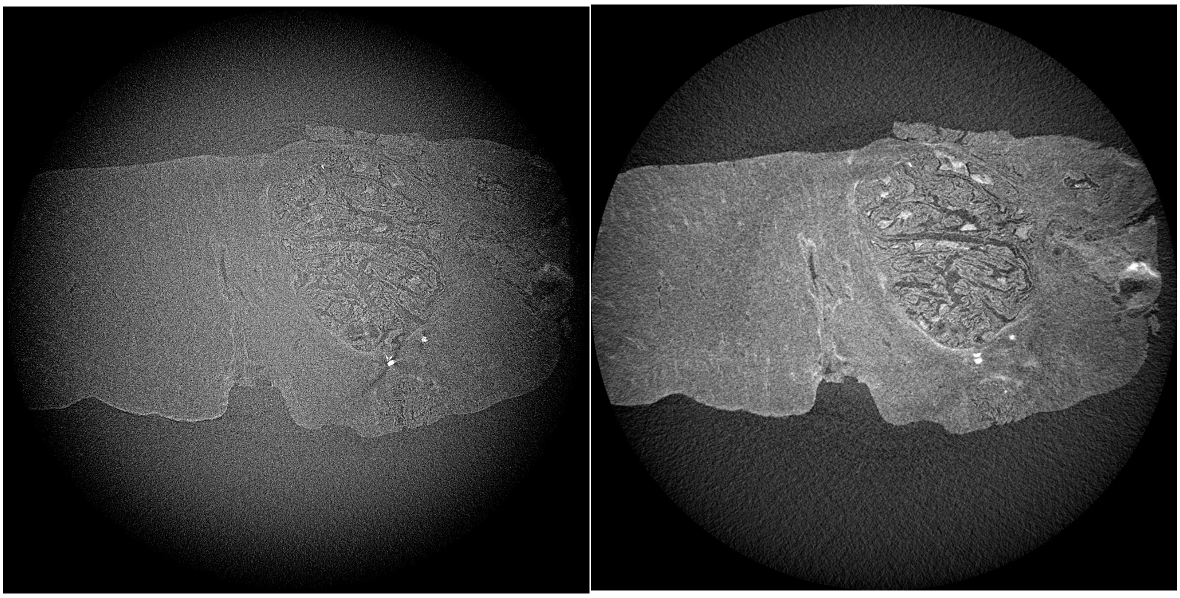

Supplement: Supplementary file 1 — Supplementary Information. [file 41598_2026_61069_MOESM1_ESM.pdf]
